# First Assessment for the Presence of Phlebotomine Vectors in Bavaria, Southern Germany, by Combined Distribution Modeling and Field Surveys

**DOI:** 10.1371/journal.pone.0081088

**Published:** 2013-11-18

**Authors:** Simone Haeberlein, Dominik Fischer, Stephanie Margarete Thomas, Ulrike Schleicher, Carl Beierkuhnlein, Christian Bogdan

**Affiliations:** 1 Mikrobiologisches Institut - Klinische Mikrobiologie, Immunologie und Hygiene, Friedrich-Alexander-Universität (FAU) Erlangen-Nürnberg and Universitätsklinikum Erlangen, Erlangen, Germany; 2 Department of Biogeography, University of Bayreuth, Bayreuth, Germany; Technion-Israel Institute of Technology Haifa 32000 Israel., Israel

## Abstract

Leishmaniasis is caused by protozoa of the genus *Leishmania* and transmitted by sand flies from mammalian reservoirs to humans. In recent years, a northward spread of *L. infantum* from highly endemic Mediterranean countries into previously non-endemic Central European areas has been suspected based on presumed sporadic cases of autochthonous leishmaniasis. Here, we investigated whether sand flies are prevalent in Bavaria in Southern Germany, a federal state in which autochthonous cases have previously been reported. Considering the present and future climatic conditions, we determined whether Bavaria is suitable for five sand fly species with assumed spreading tendencies towards Central Europe: *Phlebotomus ariasi*, *P. neglectus*, *P. perfiliewi* and *P. perniciosus* that are known vectors for *Leishmania* in Europe, and *P. mascittii*, a suspected but not proven vector. Within Bavaria we defined sampling regions based on their climatic suitability and their spatial distance to the sites of the autochthonous cases and/or to areas of reported sand fly detection in states adjacent to Bavaria. At 155 locations in 7 sampling regions, CDC light traps were placed during 38 nights in the summers of 2009 and 2010, resulting in 202 trap-nights. All traps were negative for sand flies. The results suggest that Bavaria is not yet endemic for sand flies, but do not exclude the possibility of sporadic cases of autochthonous human or zoonotic *Leishmania* infections. This study, which combined methodological approaches from different disciplines, serves as reference for future surveys and risk analyses of sand flies and leishmaniasis in so far non-endemic areas of Europe.

## Introduction

The establishment and autochthonous transmission of a vector-borne infectious disease in a formerly non-endemic region requires the presence of (1) the pathogen, (2) reservoir hosts, and (3) a competent vector. Protozoan parasites of the genus *Leishmania* are transmitted by sand flies and cause cutaneous, mucocutaneous or visceral leishmaniasis in tropical and subtropical countries worldwide. The species *Leishmania* (L.) *infantum*, which is highly prevalent in Mediterranean countries, is in Europe mainly transmitted by *Phlebotomus* (P.) *perniciosus, P. neglectus* and *P. ariasi* and accounts for cutaneous or visceral leishmaniasis in humans and animals [1,2]. The pathogen is regularly imported into Germany, presently a non-endemic country, by infected travellers and animals, especially dogs. Human leishmaniasis is not notifiable in Germany, but based on incomplete voluntary recordings the prevalence of all forms of leishmaniasis in Germany is probably in the range of several hundred cases per year [3,4]. It is estimated that about 20,000 dogs, which became infected with *Leishmania* during previous stays in endemic countries, are currently living in Germany [4–6]. Thus, it is likely that the only limitation to the transmission of leishmaniasis between various hosts is the absence of a phlebotomine vector in Germany. Since temperature constraints are known to limit the distribution of sand fly species, their occurrence at higher latitudes is presently restricted. However, increasing temperatures as a consequence of climate change are expected to promote the spread of sand flies into presently non-endemic regions [7,8]. This has already been documented in several Central European countries (e.g. Switzerland, Austria and Hungary) where sand flies have recently been recorded [9–11]. In Germany, sand flies of the species *P. mascittii*, a suspected but not proven vector for *L. infantum*, and of the species *P. perniciosus* were recently found along the Rhine valley in the Federal State of Baden-Württemberg in the south-west of Germany [12,13]. In the neighboring Federal State of Bavaria in the south-east of Germany, one case of autochthonous human leishmaniasis and three cases of autochthonous canine and equine leishmaniasis were reported from 1991 to 2000 [14–16]. These observations not only suggest the prevalence of infected sand flies in Bavaria, but also clearly underscore the need for a systematic survey for sand flies in this largest federal state of Germany, surrounded by regions with documented presence of sand flies.

Based on previously documented cases of autochthonous leishmaniasis in Southern Germany, we hypothesize that sand flies might already exist in Bavaria. In the present study we evaluated the current risk for the establishment of sand flies and leishmaniasis in Bavaria by integrating two methodological approaches: (1) Modeling of the current and future (2011 to 2040) climate-derived suitability for the establishment of sand fly species in Bavaria based on bioclimatic envelopes; and (2) a field survey on the prevalence of sand flies in the assumed climatically most favored regions of Bavaria.

## Materials and Methods

### Modeling the climatic suitability for *Phlebotomus* species

Climate and especially temperature is considered as the main habitat factor for sand fly establishment [7,8]. To this end, we used species distribution models that were based on a maximum-entropy approach [17] to refer bioclimatic variables [18] to locations in Europe that are currently endemic for various sand fly species (*P. ariasi*, *P. mascittii*, *P. neglectus*, *P. perfiliewi* and *P. perniciosus*). First, we incorporated 19 bioclimatic variables, including all available and biologically meaningful variables that may be crucial for e.g. species activity phase or possible conditions during diapause. From those, the most relevant variables explaining sand fly occurrences were detected via Jackknife test. This test measures a variable`s contribution for the model result if the variable is used in isolation or in a set with other variables (multivariate procedure). Detailed information concerning variables´ contribution, model input and runs as well as test procedures and quality criteria has been reported previously [19,20]. In short, models showed high performance power [19] which enabled us to extrapolate the detected bioclimatic suitability to the current bioclimatic situation in Bavaria for each species. In order to determine potential future suitable areas for sand fly establishment in Bavaria, we applied climate change projections obtained by the Regional Climate Model COSMO-CLM [21] for the time-frame 2011 to 2040 and the A1B emission scenario. In this second step, the determined preferred bioclimatic niche was related to the expected future climatic conditions. Model output delivers values of climatic suitability (for the current and future situation) that are theoretically ranging from 0 (completely unsuitable) up to 1 (completely suitable). Based on these values, five suitability classes were defined that reflect the suitability of an area for the establishment of each species (low: 0.0 - 0.15, rather low: 0.16 - 0.3, moderate: 0.31 - 0.45, rather high: 0.45 - 0.6, high: > 0.6).

### Sampling regions for sand fly trapping

The field survey was conducted in Bavaria in sampling regions that were defined as areas where at least two of the four epidemiological and climatic parameters described below were fulfilled:

1. Locations of four previous cases of human, equine and canine autochthonous leishmaniasis in Bavaria, where neither the patients nor their mothers have ever stayed in a *Leishmania*-endemic area and congenital transmission has been vigorously ruled out by diagnostic methods at least in the human case [14–16], suggesting a sporadic occurrence of *Leishmania*-infected sand flies;
2. occurrence of sand flies in states that are adjacent to Bavaria, assuming the geographical proximity would favor sand fly migration across border zones of Bavaria (Naucke TJ, personal communication) [10,12];
3. current climatic suitability in Bavaria for the five biogeographically most relevant sand fly species with expected spreading tendencies (*P. ariasi*, *P. mascittii*, *P. neglectus*, *P. perfiliewi* and *P. perniciosus*). Although *P. mascittii* is not yet a proven vector, its distribution is modeled due to its presence in Baden-Württemberg (Germany) and Carinthia (Austria), regions that are close to Bavaria;
4. future climatic suitability for sand fly establishment in Bavaria for the foreseeable future (projected time-frame 2011 to 2040; A1B emission scenario). To fulfill criteria 3 or 4, the value of climatic suitability of an area had to be above 0.3 which indicates at least moderate climatic suitability.

### Field survey

Presence of phlebotomine vectors was assessed by using CDC New Standard Miniature Light Traps (model 1012; John W. Hock Company, Gainesville, Florida, USA). Traps were fixed one meter above the ground and were preferentially set close to human dwellings, especially on farms close to or inside animal shelters or in old barns with non-concreted substrate, humid and organic soil cover based on reported habitat preferences of sand flies [9,13]. Traps were run overnight and collected the following morning. Each location was probed with one or two traps per night. Night temperature, wind and rain conditions, GPS coordinates, as well as characteristics of the trap location (e.g. material of the building and substrate) and presence of domestic animals were recorded to provide basic data about site conditions. Night temperatures were mostly above 12°C. Captured insects were stored in 70% ethanol or frozen at -20°C for later morphological examination using an inverted microscope (Axiovert 40C; Carl Zeiss, Goettingen, Germany) at a magnification of 100×. The field survey was approved and supported by the ‘Bavarian State Ministry of the Environment and Public Health’ and the ‘Bavarian Health and Food Safety Authority’, No 08/18.

## Results

### Sampling regions for sand fly trapping

**Figure 1:**
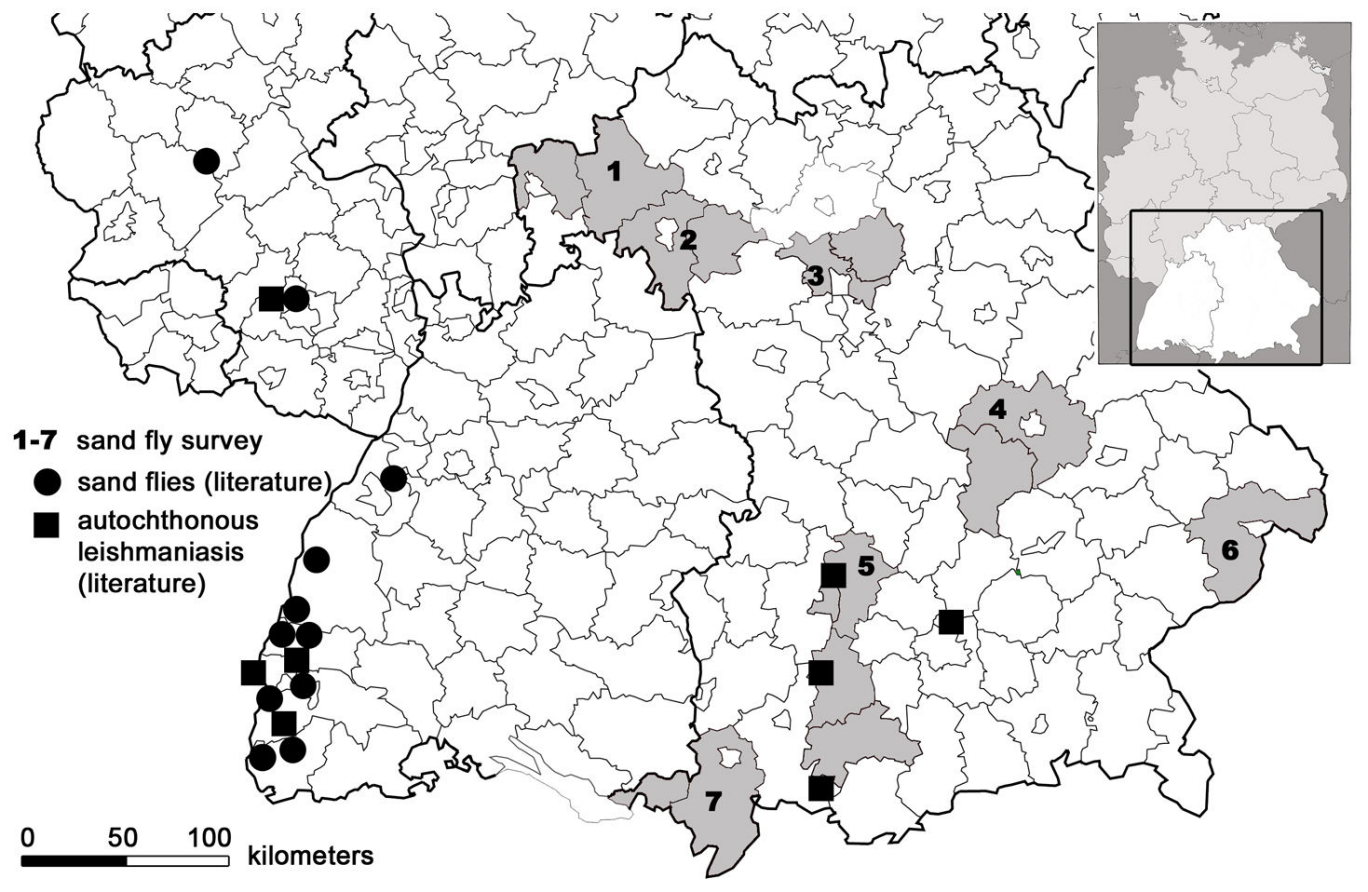
Map of southern Germany with sampling regions of the sand fly survey in the years 2009 and 2010. The insert shows a map of Germany with the Federal States of Bavaria and Baden-Württemberg highlighted. CDC light traps for sand fly trapping were put up in seven sampling areas in Bavaria (indicated by the numbers 1-7 and shaded in gray) which were allocated to the following administrative districts: 1, Aschaffenburg/Main-Spessart; 2, Würzburg/Kitzingen; 3, Erlangen-Höchstadt/Forchheim; 4, Regensburg/Kelheim; 5, Aichach-Friedberg/Landsberg am Lech/Weilheim-Schongau; 6, Passau; 7, Lindau am Bodensee/Oberallgäu. Sites of reported cases of autochthonous leishmaniasis in Bavaria and Baden-Württemberg and some of the reported sites of sand flies in Baden-Württemberg are marked [12,14–16].

According to the selection criteria, seven sampling regions were selected for the survey of sand flies (Figure 1). These regions are representing the administrative districts of Bavaria listed in Table **1**. Each sampling region fulfilled at least two of the following selection criteria: vicinity to reported cases of autochthonous leishmaniasis, vicinity to recently reported sand fly records, current climatic suitability for sand fly occurrence and/or future climatic suitability. 

**Table 1:** Survey of the prevalence of sand flies in Bavaria (southern Germany) in the years 2009 and 2010.

**Sampling region^a^**	**Administrative districts**	**Number of trap-nights (number of localities) per period^b^**				
		**2009**		**2010**		**TOTAL**
		06/15-07/15	07/16-08/31	06/15-07/15	07/16-08/31	
1	Aschaffenburg/ Main-Spessart	-	-	-	16 (12)	16 (12)
2	Würzburg/Kitzingen	-	-	8 (4)	11 (6)	19 (10)
3	Erlangen-Höchstadt/ Forchheim	17 (9)	6 (4)	21 (18)	9 (8)	53 (35^d^)
4	Regensburg/ Kelheim	-	7 (4)	9 (9)	17 (17)	33 (29^d^)
5	Aichach-Friedberg/ Landsberg am Lech/ Weilheim-Schongau	-	8 (7)	-	24 (20)	32 (27)
6	Passau	-	5 (4)	-	24 (22)	29 (26)
7	Lindau am Bodensee/ Oberallgäu	-	-	-	5 (4)	5 (4)
-	Others^c^	5 (5)	10 (7)	-	-	15 (12)
	**TOTAL**	**22 (14)**	**36 (26)**	**38 (31)**	**106 (89)**	**202** (**155** ^d^)

^a^ Sampling regions were defined on the basis of epidemiological parameters (previously published cases of autochthonous cases of leishmaniasis in Bavaria, and proven sand fly occurrence in states adjacent to Bavaria) and climate parameters (current and future climatic suitability for the upcoming time-period 2011 to 2040 in Bavaria for five sand fly species with expected spreading tendencies, i.e. *P. ariasi*, *P. mascittii*, *P. neglectus*, *P. perfiliewi* and *P. perniciosus*).

^b^ CDC light traps were mounted in 2009 and 2010 during June to August overnight outside or in stables and old barns and analysed for sand flies. No sand fly was trapped.

^c^ Administrative districts: Ansbach, Ebersberg, Rosenheim, Weißenburg-Gunzenhausen

^d^ Some localities were surveyed in both years.

**Figure 2:**
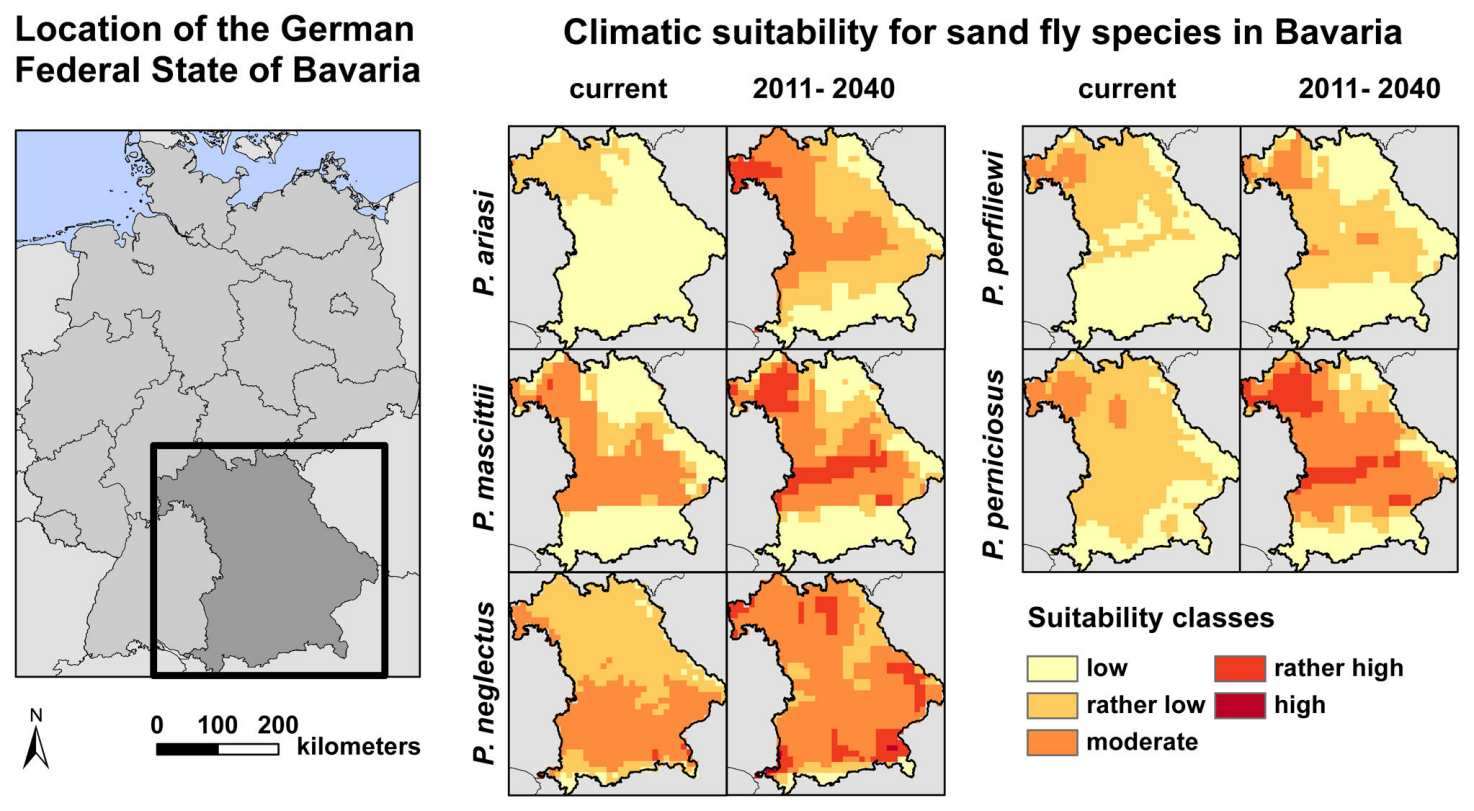
Current and projected (2011-2040) climatic suitability for five sand fly species in Bavaria (southern Germany). Currently, the climatic suitability for sand fly species is rather low or moderate in Bavaria. The projections for future climate change refer to the A1B emission scenario of greenhouse gases, expecting an economical growth in a globalized world with a balanced use of fossil and non-fossil energy resources. During the next decades it can be expected that the suitability will increase. This is true for all analyzed sand fly species. A rather high climatic suitability can be expected for the valley of the river Main, especially for Lower Franconia in the most north-western part of Bavaria, along the river Danube and for the alpine foothills.

In regions 1 to 6, modeling of the current climatic conditions identified at least moderately adequate climatic conditions for the establishment of sand flies, whereas the projected future climatic conditions in the time-frame 2011 to 2040 yielded for all seven regions “rather high” climatic suitability classes for the establishment of the sand fly species under investigation (Figure **2**). The most important main variables that were influencing the distribution of all modeled species were the mean temperature of the coldest quarter of the year, followed by the mean temperature of the warmest quarter and, to a lesser extent, also the precipitation of the coldest quarter. For *P. perniciosus*, however, the precipitation of the wettest quarter has the highest explanatory power and must be considered as major variable for the occurrence and distribution of this vector species. A detailed statistical description of the contribution of the different variables has been reported [19]. 

According to our distribution models, the north-western part of Bavaria presently shows the highest climatic suitability for four of the five sand fly species (*P. mascittii*, *P. neglectus*, *P. perfiliewi*, *P. perniciosus*). Within the following decades, favorable climatic conditions for sand flies are likely to develop in areas along the river Danube (for *P. mascittii*, *P. perniciosus*) and the alpine foothills in the South of Bavaria (for *P. neglectus*) (Figure **2**). The selection of the sampling region 5 (Figure **1**) was additionally based on reported cases of autochthonous leishmaniasis in a child, two dogs and a horse in the Bavarian administrative districts of Ostallgäu, Landsberg am Lech and Augsburg [14–16], whereas the sampling regions 1, 6 and 7 (Figure **1**) were particularly chosen for their vicinity to Austria and to the German Federal State of Baden-Württemberg, where sand flies have recently been reported (Naucke TJ, personal communication) [10,12].

### Prevalence of sand flies in Bavaria

In the years 2009 and 2010, insect sampling was undertaken with 202 CDC light traps set up during 38 nights (202 trap-nights) in 155 different localities in Bavaria from the mid of June until end of August when warm weather conditions are thought to favor sand fly activity [12] (Table **1**; table **S1** **for georeferences**). Because particularly warm nights were chosen, the trapping was done periodically and not consecutively within this time-period. Most traps were set up in the warmest season from the second half of July to August. 187 traps were located inside the seven sampling regions. In addition, for control purposes we placed a number of traps outside the sampling regions. In 2009, only 58 traps in 17 different nights were set up in 40 localities because of a relatively cold and rainy summer in Bavaria, with a mean temperature in July of 18.1°C in 2009 compared to 20.2°C in 2010 [22]. The low temperatures in 2009 were assumed to counteract activity of sand flies considering that the species under investigation need a mean temperature of at least 18 to 20°C during the warmest month to become active [23]. In 2010, 144 traps in 21 different nights were placed in 120 localities. All traps contained a number of 50 to 200 insects, with the majority being dipterans, including different mosquito species (e.g. *Culex* spp.) and numerous specimens of the genus *Psychoda*, which together with the genus *Phlebotomus* belongs to the Psychodidae family. However, after microscopical examination of all captured insects we could not detect one single sand fly. This even includes the districts of Aichach-Friedberg, Landsberg am Lech and Weilheim-Schongau that are close to the sites of previous sporadic cases of autochthonous leishmaniasis [14–16] and had therefore suggested the presence of sand flies.

## Discussion

In this study we tested the presence of sand flies in the Federal State of Bavaria in south-eastern Germany. The hypothesis was based on sporadic autochthonous cases of human and animal leishmaniasis in Bavaria, on the reported prevalence of sand flies in neighboring states (Baden-Württemberg, Switzerland and Austria), and on the fact that within Germany the southern part of the country offers the most promising climatic conditions for thermophilic sand flies. In this survey, no sand flies could be recorded in the selected sampling areas. Missing positive records of a species do not prove its absence. However, we are convinced that the combined approach of modeling and sampling is of sufficient validity for two reasons: First, the applied models hold a high performance power [19]. Second, a comparable procedure provided the first evidence for sand flies in Austria [10] in the formerly non-endemic region of Carinthia that had previously been identified to be suitable for sand flies by climatic modeling efforts [23]. In consequence, we conclude that sand flies are most likely not yet endemic in the south-eastern part of Germany. According to our modeling, climatic conditions in Bavaria are currently only moderately appropriate for sand fly habitats (suitability value 0.31-0.45) (Figure **2**). Thus, although sporadic emergence of these vectors cannot be firmly excluded, its likelihood is still low.

In general, correlation of species occurrences with climatic variables can explain the current sand fly distribution and allows to project potential responses (e.g. range expansions or shifts) to climatic changes [24–26]. For the purpose of this study, bioclimatic variables were used as they are considered to deliver meaningful explanations for biological processes of species. The mean temperature of the coldest quarter exhibits the highest explanatory power for sand fly occurrences. This is in accordance with the fact that sand flies overwinter in diapause to minimize environmental stressors and emphasizes the significance of climatic constraints throughout the year [27,28]. Following these considerations reliable species distribution models should not only be based on variables affecting species activity phases. We took this explicitly into account when we decided upon the parameters for selecting the sampling regions of the field survey.

Determining climatic or environmental suitability for the presence of vector species, as performed in this study, is not the only way to identify risk areas of transmission. In endemic areas of leishmaniasis in Southern Europe, correlative niche models successfully predicted risk areas of transmission [29,30]. Such models can be trained or tested with seroprevalence data. Another possibility for risk determination is the mapping of model results for the basic reproductive number R0 if data on sand fly abundance are available [31]. This has been demonstrated for canine leishmaniasis in Southern France [32]. The spatial mapping of R0 illustrates, in which areas an increase, decrease or persistence of cases can be expected. However, such detailed information is only available for endemic regions and cannot be transferred to non-endemic regions such as Bavaria. Due to these limitations, the definition of focus regions was not exclusively based on bioclimatic suitability of the vectors. In order to achieve geographically representative results, locations of previous cases of autochthonous leishmaniasis were included as well as regions that border areas with established sand fly foci.

The absence of sand flies during the years 2009 and 2010 was unexpected in the light of previously reported autochthonous cases of leishmaniasis [14–16]. However, from our field study in 2009 and 2010, we cannot exclude a periodical presence of sand flies in Bavaria in earlier years following sporadic introduction. A similar explanation can hold true for a recent study performed in Hungary that failed to trap sand flies at a location of previously reported autochthonous leishmaniasis in dogs [33], although sand flies have been recorded in the same year in other regions in Hungary [11]. With respect to the possibility that non-sand fly transmission modes might underlie the reported four cases of autochthonous leishmaniasis in Bavaria [14–16], the known medical histories and the performed diagnostic procedures allow to exclude congenital transmission at least in the human case [15] and to a lesser extent also in the equine case [16]. In light of the recently reported vertical transmission of *L. infantum* in naturally infected dogs [34,35], including one suspected case in Germany, this way of transmission should be given increased attention in future studies. Whether other arthropods such as the brown dog tick (*Rhipicephalus sanguineus*), which was recently found positive for *Leishmania* DNA in an endemic Mediterranean country [36,37], function as vectors for canine leishmaniasis, also needs further investigation. 

With respect to local introduction of vectors, it is still questionable whether humans actively assist in the spread of sand flies. In addition, sand flies usually do not respond to environmental changes with rapid range expansions due to their limited ability to fly [27]. Assuming that sand fly distribution is solely related to active natural spreading without passive transports to climatically suitable areas, the range expansions of sand flies in Central Europe happen on time-scales of several years or decades but not months [19,20]. Nevertheless, with regard to future projections, we illustrate that climate change is likely to support the establishment of sand fly species already in the near future (time-frame: 2011 to 2040, A1B scenario) in certain districts of Bavaria.

We see this regional case study as an example for other parts of Europe. Of note, the first autochthonous human infections with Toscana virus, which is endemic in Mediterranean countries and also transmitted by sand flies, have recently been reported in south-western Germany [38]. The first record of this pathogen north of the Alps provides further evidence for the northward expansion of sand flies and sand fly-borne diseases. 

## Conclusions

By combining species distribution models and field surveys, we conclude that at present south-eastern Germany is unlikely to be an epidemiologically relevant endemic area for sand flies. Thus, we consider the current risk for humans to acquire autochthonous leishmaniasis as low. This may change in the future if climatic developments further facilitate the spread of phlebotomine vectors to Germany and other non-endemic areas of Central Europe as indicated by climatic projections combined with species distribution models. Future surveys on the presence of sand flies are warranted to detect the potential establishment of leishmaniasis in Germany and to initiate control measures in time. The results of the current study serve as an important reference for forthcoming analyses and surveillance activities. 

## Supporting Information

Table S1 **Georeferences and mounting date of each CDC light trap that was run overnight within 7 sampling regions in Bavaria, southern Germany, during the field survey in the years 2009 and 201.**
(DOC)
