# Supplementary material for: First Assessment for the Presence of Phlebotomine Vectors in Bavaria, Southern Germany, by Combined Distribution Modeling and Field Surveys
Source: PLoS One. 2013 Nov 18;8(11):e81088. doi: 10.1371/journal.pone.0081088 (PMC3832422; doi:10.1371/journal.pone.0081088)
Supplement: Table S1 — Georeferences and mounting date of each CDC light trap that was run overnight within 7 sampling regions in Bavaria, southern Germany, during the field survey in the years 2009 and 201. (DOC) [file pone.0081088.s001.doc]

**Supplemental table S1. Georeferences and mounting date of each CDC light trap that was run overnight within 7 sampling regions in Bavaria, southern Germany, during the field survey in the years 2009 and 2010.**

| **Sampling region** | **Date** | **Latitude** | **Longitude** |
| --- | --- | --- | --- |
| 1 | 2010-07-30 | 50° 0'14.77"N | 9°13'40.57"E |
| 1 | 2010-07-30 | 50° 0'14.77"N | 9°13'40.59"E |
| 1 | 2010-07-30 | 50° 1'2.15"N | 9°14'57.87"E |
| 1 | 2010-07-30 | 50° 2'36.07"N | 9°15'15.69"E |
| 1 | 2010-07-30 | 50° 2'55.65"N | 9°15'38.79"E |
| 1 | 2010-07-30 | 50° 6'3.75"N | 9°14'46.63"E |
| 1 | 2010-07-30 | 50° 7'52.98"N | 9°13'59.39"E |
| 1 | 2010-07-30 | 50° 8'3.56"N | 9°13'7.02"E |
| 1 | 2010-08-28 | 49°49'34.98"N | 9°31'47.82"E |
| 1 | 2010-08-28 | 49°51'31.43"N | 9°31'8.83"E |
| 1 | 2010-08-28 | 49°51'31.45"N | 9°31'8.81"E |
| 1 | 2010-08-28 | 49°51'40.20"N | 9°34'24.87"E |
| 1 | 2010-08-28 | 49°52'10.62"N | 9°34'48.89"E |
| 1 | 2010-08-28 | 49°55'42.38"N | 9°30'12.86"E |
| 1 | 2010-08-28 | 49°56'42.24"N | 9°28'45.75"E |
| 1 | 2010-08-28 | 49°58'51.12"N | 9°27'44.46"E |
| 2 | 2010-07-10 | 49°45'14.19"N | 10° 1'44.88"E |
| 2 | 2010-07-10 | 49°45'14.20"N | 10° 1'44.88"E |
| 2 | 2010-07-10 | 49°45'13.86"N | 10° 0'28.13"E |
| 2 | 2010-07-10 | 49°42'33.98"N | 10° 0'49.26"E |
| 2 | 2010-07-10 | 49°42'33.98"N | 10° 0'49.26"E |
| 2 | 2010-07-10 | 49°45'11.91"N | 9°56'36.16"E |
| 2 | 2010-07-10 | 49°45'13.92"N | 9°56'19.27"E |
| 2 | 2010-07-10 | 49°41'33.03"N | 10°10'15.50"E |
| 2 | 2010-08-07 | 49°43'53.57"N | 10° 2'0.98"E |
| 2 | 2010-08-07 | 49°43'53.66"N | 10° 2'4.16"E |
| 2 | 2010-08-07 | 49°42'47.69"N | 10° 4'5.50"E |
| 2 | 2010-08-07 | 49°40'40.09"N | 10° 8'40.15"E |
| 2 | 2010-08-07 | 49°40'14.64"N | 10° 6'12.17"E |
| 2 | 2010-08-20 | 49°38'34.67"N | 10° 7'34.49"E |
| 2 | 2010-08-20 | 49°38'34.67"N | 10° 7'34.49"E |
| 2 | 2010-08-20 | 49°38'58.64"N | 10° 4'37.91"E |
| 2 | 2010-08-20 | 49°38'58.64"N | 10° 4'37.91"E |
| 2 | 2010-08-20 | 49°37'37.12"N | 9°59'59.07"E |
| 2 | 2010-08-20 | 49°37'37.12"N | 9°59'59.07"E |
| 3 | 2009-06-16 | 49°37'37.06"N | 10°56'18.78"E |
| 3 | 2009-06-16 | 49°38'34.01"N | 10°53'38.51"E |
| 3 | 2009-06-16 | 49°38'44.82"N | 10°53'30.29"E |
| 3 | 2009-06-16 | 49°38'42.11"N | 10°53'40.49"E |
| 3 | 2009-06-16 | 49°38'48.19"N | 10°52'23.41"E |
| 3 | 2009-06-16 | 49°38'13.45"N | 11°18'57.06"E |
| 3 | 2009-06-16 | 49°38'13.45"N | 11°18'57.06"E |
| 3 | 2009-06-16 | 49°38'13.45"N | 11°18'57.06"E |
| 3 | 2009-06-30 | 49°38'17.50"N | 10°51'1.92"E |
| 3 | 2009-06-30 | 49°37'46.60"N | 10°52'40.55"E |
| 3 | 2009-06-30 | 49°38'13.73"N | 10°52'13.03"E |
| 3 | 2009-06-30 | 49°37'50.88"N | 10°52'35.72"E |
| 3 | 2009-06-30 | 49°39'0.76"N | 10°49'32.84"E |
| 3 | 2009-06-30 | 49°38'17.50"N | 10°51'1.92"E |
| 3 | 2009-06-30 | 49°38'5.97"N | 10°49'5.75"E |
| 3 | 2009-06-30 | 49°37'56.02"N | 10°49'14.89"E |
| 3 | 2009-07-04 | 49°35'27.49"N | 11° 2'22.16"E |
| 3 | 2009-07-17 | 49°39'2.65"N | 11°15'49.92"E |
| 3 | 2009-07-17 | 49°31'36.07"N | 11°49'6.68"E |
| 3 | 2009-07-21 | 49°36'35.86"N | 11° 7'0.48"E |
| 3 | 2009-07-21 | 49°36'35.86"N | 11° 7'0.48"E |
| 3 | 2009-07-27 | 49°38'13.45"N | 11°18'57.06"E |
| 3 | 2009-07-27 | 49°38'13.45"N | 11°18'57.06"E |
| 3 | 2010-06-15 | 49°36'38.86"N | 11° 3'24.68"E |
| 3 | 2010-06-15 | 49°38'32.42"N | 11° 2'7.55"E |
| 3 | 2010-06-15 | 49°39'54.32"N | 11° 3'57.92"E |
| 3 | 2010-06-15 | 49°39'30.60"N | 11° 5'30.91"E |
| 3 | 2010-06-15 | 49°38'27.02"N | 11°4'17.65"E |
| 3 | 2010-06-29 | 49°38'23.52"N | 10°51'2.52"E |
| 3 | 2010-06-29 | 49°35'51.60"N | 10°49'39.67"E |
| 3 | 2010-06-29 | 49°35'31.38"N | 10°53'44.44"E |
| 3 | 2010-06-29 | 49°32'52.71"N | 10°50'30.44"E |
| 3 | 2010-06-29 | 49°41'20.66"N | 10°52'36.66"E |
| 3 | 2010-06-29 | 49°42'22.95"N | 10°51'28.71"E |
| 3 | 2010-06-29 | 49°42'59.53"N | 10°49'2.64"E |
| 3 | 2010-07-02 | 49°38'0.88"N | 10°49'12.88"E |
| 3 | 2010-07-02 | 49°38'12.91"N | 10°50'56.05"E |
| 3 | 2010-07-02 | 49°39'0.64"N | 10°49'33.56"E |
| 3 | 2010-07-02 | 49°39'22.60"N | 10°48'33.31"E |
| 3 | 2010-07-13 | 49°36'38.34"N | 11° 3'26.33"E |
| 3 | 2010-07-13 | 49°37'46.87"N | 11° 3'46.57"E |
| 3 | 2010-07-13 | 49°39'56.79"N | 11° 6'44.28"E |
| 3 | 2010-07-13 | 49°40'23.45"N | 11°10'12.39"E |
| 3 | 2010-07-13 | 49°39'7.52"N | 11°10'18.94"E |
| 3 | 2010-08-07 | 49°37'51.04"N | 10°53'6.78"E |
| 3 | 2010-08-07 | 49°37'8.84"N | 10°46'20.61"E |
| 3 | 2010-08-07 | 49°36'59.05"N | 10°44'45.29"E |
| 3 | 2010-08-07 | 49°36'37.21"N | 10°43'53.14"E |
| 3 | 2010-08-07 | 49°36'16.60"N | 10°40'17.00"E |
| 3 | 2010-08-07 | 49°36'11.74"N | 10°40'23.83"E |
| 3 | 2010-08-07 | 49°37'30.69"N | 10°41'10.73"E |
| 3 | 2010-08-07 | 49°37'42.69"N | 10°43'4.74"E |
| 3 | 2010-08-07 | 49°37'46.39"N | 10°44'15.17"E |
| 4 | 2009-07-30 | 48°58'13.51"N | 12°20'0.10"E |
| 4 | 2009-07-30 | 48°57'37.19"N | 12°22'9.52"E |
| 4 | 2009-07-30 | 48°57'15.77"N | 12°24'57.48"E |
| 4 | 2009-07-30 | 48°56'54.82"N | 12°24'51.84"E |
| 4 | 2009-07-30 | 48°57'0.72"N | 12°24'35.53"E |
| 4 | 2009-07-30 | 48°57'26.35"N | 12°14'19.75"E |
| 4 | 2009-07-30 | 48°57'35.35"N | 12°16'43.14"E |
| 4 | 2010-07-10 | 49°0'29.09"N | 11°57'37.80"E |
| 4 | 2010-07-10 | 48°58'51.51"N | 11°57'39.11"E |
| 4 | 2010-07-10 | 48°58'7.97"N | 11°58'55.14"E |
| 4 | 2010-07-10 | 48°53'34.75"N | 12° 0'41.49"E |
| 4 | 2010-07-10 | 48°53'15.37"N | 12° 3'44.61"E |
| 4 | 2010-07-10 | 48°53'5.44"N | 12° 6'47.45"E |
| 4 | 2010-07-10 | 48°53'37.54"N | 12° 8'15.24"E |
| 4 | 2010-07-10 | 48°54'42.08"N | 12° 9'37.04"E |
| 4 | 2010-07-10 | 48°55'19.80"N | 12°11'2.12"E |
| 4 | 2010-08-04 | 48°58'11.63"N | 12°14'32.29"E |
| 4 | 2010-08-04 | 48°57'25.33"N | 12°14'20.14"E |
| 4 | 2010-08-04 | 48°56'53.52"N | 12°13'10.52"E |
| 4 | 2010-08-04 | 48°55'59.07"N | 12°15'34.68"E |
| 4 | 2010-08-04 | 48°55'38.37"N | 12°15'59.94"E |
| 4 | 2010-08-04 | 48°58'32.99"N | 12°17'19.51"E |
| 4 | 2010-08-04 | 48°56'32.99"N | 12°13'15.99"E |
| 4 | 2010-08-04 | 48°57'34.23"N | 12°15'5.45"E |
| 4 | 2010-08-04 | 48°57'9.65"N | 12°10'17.96"E |
| 4 | 2010-08-20 | 48°52'49.66"N | 11°53'55.10"E |
| 4 | 2010-08-20 | 48°53'0.50"N | 11°53'1.68"E |
| 4 | 2010-08-20 | 48°51'19.64"N | 11°54'3.73"E |
| 4 | 2010-08-20 | 48°50'43.01"N | 11°55'54.63"E |
| 4 | 2010-08-20 | 48°50'17.12"N | 11°57'28.52"E |
| 4 | 2010-08-20 | 48°50'52.32"N | 11°56'45.12"E |
| 4 | 2010-08-20 | 48°50'51.06"N | 12° 0'53.61"E |
| 4 | 2010-08-20 | 48°51'31.20"N | 12° 2'58.29"E |
| 5 | 2009-07-24 | 47°58'52.82"N | 10°50'48.08"E |
| 5 | 2009-07-24 | 47°57'29.45"N | 10°52'41.27"E |
| 5 | 2009-07-24 | 47°58'50.59"N | 10°54'10.89"E |
| 5 | 2009-07-25 | 47°54'40.61"N | 10°51'25.36"E |
| 5 | 2009-07-25 | 47°46'23.84"N | 10°56'12.19"E |
| 5 | 2009-07-25 | 47°54'42.01"N | 10°51'1.08"E |
| 5 | 2009-07-25 | 47°54'28.30"N | 10°50'48.88"E |
| 5 | 2009-07-25 | 47°48'21.75"N | 10°49'10.90"E |
| 5 | 2010-07-20 | 48°22'29.11"N | 10°45'32.89"E |
| 5 | 2010-07-20 | 48°21'26.17"N | 10°44'46.39"E |
| 5 | 2010-07-20 | 48°20'32.15"N | 10°43'57.86"E |
| 5 | 2010-07-20 | 48°18'44.18"N | 10°42'35.11"E |
| 5 | 2010-07-20 | 48°17'31.96"N | 10°42'27.00"E |
| 5 | 2010-07-20 | 48°16'30.97"N | 10°42'12.39"E |
| 5 | 2010-07-20 | 48°15'17.00"N | 10°44'11.19"E |
| 5 | 2010-07-20 | 48°12'57.54"N | 10°50'13.50"E |
| 5 | 2010-07-20 | 48° 9'51.55"N | 10°48'30.36"E |
| 5 | 2010-07-20 | 48° 8'53.85"N | 10°48'22.67"E |
| 5 | 2010-07-24 | 47°44'25.84"N | 10°57'23.87"E |
| 5 | 2010-07-24 | 47°44'41.93"N | 10°56'49.56"E |
| 5 | 2010-07-24 | 47°46'36.84"N | 10°55'47.42"E |
| 5 | 2010-07-24 | 47°45'49.60"N | 10°58'48.05"E |
| 5 | 2010-07-24 | 47°45'0.58"N | 10°58'35.04"E |
| 5 | 2010-07-24 | 47°42'56.88"N | 10°54'26.82"E |
| 5 | 2010-07-24 | 47°44'22.09"N | 10°52'38.96"E |
| 5 | 2010-08-14 | 48°15'44.81"N | 11° 0'52.03"E |
| 5 | 2010-08-14 | 48°15'45.12"N | 11° 1'3.78"E |
| 5 | 2010-08-14 | 48°16'12.05"N | 11° 1'14.76"E |
| 5 | 2010-08-14 | 48°15'24.88"N | 11° 5'39.73"E |
| 5 | 2010-08-14 | 48°13'19.14"N | 11° 7'29.58"E |
| 5 | 2010-08-14 | 48°11'21.55"N | 11°12'30.37"E |
| 5 | 2010-08-14 | 48°11'8.37"N | 11°12'47.11"E |
| 6 | 2009-07-31 | 48°19'9.30"N | 13°16'20.78"E |
| 6 | 2009-07-31 | 48°18'8.78"N | 13°13'36.95"E |
| 6 | 2009-07-31 | 48°19'9.60"N | 13°16'27.99"E |
| 6 | 2009-07-31 | 48°20'44.70"N | 13°18'34.17"E |
| 6 | 2009-07-31 | 48°18'32.65"N | 13°15'32.26"E |
| 6 | 2010-08-02 | 48°33'33.08"N | 13°14'22.23"E |
| 6 | 2010-08-02 | 48°31'53.01"N | 13°16'29.99"E |
| 6 | 2010-08-02 | 48°30'51.94"N | 13°16'33.64"E |
| 6 | 2010-08-02 | 48°30'0.33"N | 13°16'8.92"E |
| 6 | 2010-08-02 | 48°28'58.83"N | 13°17'51.72"E |
| 6 | 2010-08-02 | 48°26'34.02"N | 13°16'20.95"E |
| 6 | 2010-08-02 | 48°26'19.81"N | 13°19'18.68"E |
| 6 | 2010-08-10 | 48°25'56.53"N | 13°17'41.15"E |
| 6 | 2010-08-10 | 48°26'5.16"N | 13°14'50.67"E |
| 6 | 2010-08-10 | 48°25'40.86"N | 13°12'53.81"E |
| 6 | 2010-08-10 | 48°23'49.60"N | 13°14'57.93"E |
| 6 | 2010-08-10 | 48°23'48.66"N | 13°17'4.45"E |
| 6 | 2010-08-14 | 48°33'35.29"N | 13°17'55.10"E |
| 6 | 2010-08-14 | 48°32'13.52"N | 13°18'37.10"E |
| 6 | 2010-08-14 | 48°31'42.36"N | 13°20'1.63"E |
| 6 | 2010-08-14 | 48°30'55.64"N | 13°20'19.08"E |
| 6 | 2010-08-14 | 48°30'38.67"N | 13°21'17.04"E |
| 6 | 2010-08-14 | 48°29'34.97"N | 13°21'26.82"E |
| 6 | 2010-08-14 | 48°31'3.89"N | 13°22'25.08"E |
| 6 | 2010-08-21 | 48°22'14.74"N | 13°10'58.76"E |
| 6 | 2010-08-21 | 48°20'38.69"N | 13°16'14.59"E |
| 6 | 2010-08-21 | 48°18'43.05"N | 13°10'59.73"E |
| 6 | 2010-08-21 | 48°19'52.21"N | 13°12'3.38"E |
| 6 | 2010-08-21 | 48°18'23.01"N | 13° 8'40.02"E |
| 7 | 2010-07-31 | 47°34'43.82"N | 10°11'16.40"E |
| 7 | 2010-07-31 | 47°36'32.47"N | 10° 5'39.23"E |
| 7 | 2010-07-31 | 47°36'0.04"N | 10°11'34.08"E |
| 7 | 2010-07-31 | 47°31'17.72"N | 10°13'38.96"E |
| 7 | 2010-07-31 | 47°32'5.60"N | 10°17'16.12"E |
| Others | 2009-07-03 | 48° 1'24.28"N | 11°59'33.97"E |
| Others | 2009-07-03 | 47°56'21.14"N | 12° 0'23.16"E |
| Others | 2009-07-03 | 47°54'25.94"N | 12° 4'49.93"E |
| Others | 2009-07-04 | 49°25'10.32"N | 10°37'9.11"E |
| Others | 2009-07-05 | 49°27'1.87"N | 11° 4'49.73"E |
| Others | 2009-08-04 | 49° 3'22.57"N | 10°47'34.62"E |
| Others | 2009-08-04 | 49° 3'12.96"N | 10°47'31.81"E |
| Others | 2009-08-04 | 49° 2'34.01"N | 10°49'45.55"E |
| Others | 2009-08-04 | 49° 2'33.50"N | 10°49'32.81"E |
| Others | 2009-08-04 | 49° 5'14.03"N | 10°49'6.71"E |
| Others | 2009-08-04 | 49° 3'11.56"N | 10°50'32.46"E |
| Others | 2009-08-04 | 49° 3'37.26"N | 10°49'40.33"E |
| Others | 2009-08-04 | 49° 5'31.74"N | 10°47'9.96"E |
| Others | 2009-08-07 | 49°25'10.32"N | 10°37'9.11"E |
| Others | 2009-08-14 | 49°24'8.53"N | 10°35'27.46"E |
